# Developmental and aging trajectories of 40-Hz auditory steady-state responses: A systematic review across the human lifespan

**DOI:** 10.1016/j.dcn.2026.101690

**Published:** 2026-02-05

**Authors:** Aurimas Mockevičius, Danylo Machevskyi, Dariusz Majcherczyk, Inga Griškova-Bulanova

**Affiliations:** Translational Health Research Institute, Faculty of Medicine, Vilnius University, Vilnius, Lithuania

**Keywords:** Auditory steady-state response, Gamma synchronization, Excitation-inhibition balance, Aging, Developmental, Lifespan

## Abstract

Auditory steady-state responses (ASSRs) are rhythmic neural oscillations that synchronize to periodic auditory stimulation and serve as a noninvasive index of cortical network dynamics. ASSRs, particularly those at 40 Hz, have received substantial attention as sensitive markers of temporal precision, excitation–inhibition balance, and functional connectivity in the auditory cortex, and have been widely applied in translational research on neurodevelopmental and neuropsychiatric disorders. Because gamma synchronization supports key cognitive functions, including auditory temporal processing, selective attention, speech perception, and early language development, mapping its lifespan trajectory provides insight into how maturing cortical dynamics underpin cognitive development. However, despite extensive clinical use, the normative developmental and aging trajectory of gamma-range ASSRs remains unclear. This systematic review aimed to synthesize evidence on age-related differences in ASSRs measured with EEG or MEG across the human lifespan. Following PRISMA guidelines, searches were conducted in PubMed/Scopus, identifying 40 eligible studies. The findings reveal a pronounced increase in ASSR amplitude and phase-locking from infancy through adolescence, consistent with maturation of inhibitory circuitry, synaptic refinement, and myelination. In adulthood and aging, results were heterogeneous, with studies reporting preserved, diminished, or enhanced 40-Hz synchronization, reflecting diverse methodological approaches and potentially distinct neurobiological changes. Lifespan coverage across studies was uneven, with sparse data in early childhood and older adulthood, and limited longitudinal evidence. The review suggests a nonlinear trajectory characterized by developmental strengthening, adult stability, and variable age-related change. Comprehensive lifespan-spanning and longitudinal studies are needed to establish normative patterns and improve the interpretability of ASSR alterations in clinical populations.

## Highlights

• ASSRs show strong maturation from infancy to adolescence.
• Adult ASSR findings vary across studies.
• Aging effects on 40-Hz ASSRs are heterogeneous and non-linear.
• Lifespan coverage in ASSR research remains incomplete.
• More longitudinal studies are needed for normative trajectories.

## 1 Introduction

Auditory steady-state responses (ASSRs) refer to rhythmic neural activities that align with the temporal patterns of periodic auditory stimuli (Picton et al., 2003). When sounds are modulated at specific frequencies, cortical populations synchronize their activity to the stimulus rhythm, producing a measurable outcome in EEG or MEG recordings (Legget et al., 2017).

While ASSRs can be elicited across a broad range of modulation frequencies, responses within the gamma range (30–100 Hz) have attracted particular attention because they reflect fast neural synchronization that stands as an essential mechanism of perceptual binding, attention, and working memory (Başar-Eroglu et al., 1996). Within this range, 40-Hz ASSR has emerged as a particularly robust outcome, linked to the functional integrity of GABAergic and glutamatergic networks within the auditory cortex (Toso et al., 2024). Consequently, the 40-Hz ASSR has become a tool reflecting excitation–inhibition (E/I) balance (Tada et al., 2020), temporal processing abilities (Kadowaki et al., 2022), and state of functional connectivity (Du et al., 2023; Ying et al., 2015) in the human brain. It stands as an attractive output for translational research offering a stable, noninvasive, and frequency-specific index of neural synchronization (Farahani et al., 2020a, Hirano et al., 2020).

Abnormalities in ASSR strength and synchronization level have been reported in neuropsychiatric and neurodevelopmental disorders, including schizophrenia (Thuné et al., 2016, Zouaoui et al., 2023), bipolar disorder (Jefsen et al., 2022), autism spectrum disorder (Arutiunian et al., 2023, Seymour et al., 2020), Alzheimer’s disease (van Deursen et al., 2011), and corresponding animal models (Jasinskyte et al., 2025) where alterations in network structure and E/I dynamics are well documented. Furthermore, evidence suggests that ASSR may reflect cognitive abilities (Parciauskaite et al., 2021). These observations make ASSRs a valuable candidate biomarker for probing cortical network function across both health and disease.

Despite extensive research in clinical populations, the normative trajectory of gamma-range ASSRs across the human lifespan remains poorly defined. While individual studies have investigated developmental or aging effects on ASSRs, findings have not yet been systematically integrated and full picture is not clear. Without a comprehensive understanding of how ASSRs mature during typical development and evolve with aging, the specificity and interpretability of abnormalities observed in clinical samples remain uncertain. Establishing normative patterns of ASSR properties from early childhood through late adulthood is therefore crucial for distinguishing pathological alterations from age-appropriate variability. A systematic synthesis of lifespan data is needed to determine whether changes observed in clinical populations reflect delayed maturation, accelerated decline, or distinct pathophysiological mechanisms.

At the neurobiological level, the ability to generate and maintain gamma-range ASSRs depends on the coordinated activity of excitatory pyramidal neurons and inhibitory parvalbumin-positive (PV⁺) interneurons (Tada et al., 2020), which regulate the precise timing of cortical firing (Buzsáki and Wang, 2012). Developmental changes in ASSRs may thus reflect the maturation of inhibitory circuitry, synaptic refinement, and progressive myelination (Toga et al., 2006) that enhance temporal precision within auditory pathways. Conversely, age-related changes in GABAergic function (Zuppichini et al., 2024), synaptic density (Toyonaga et al., 2024), and white-matter integrity (Salat et al., 2005) may underlie a diminished capacity for neural synchronization in later life. Together, these processes suggest a potentially non-linear lifespan pattern of gamma synchronization.

Although the cellular and neurochemical bases of ASSR generation are increasingly well understood, no previous synthesis has integrated these mechanistic insights with empirical findings across different life stages. The present review aims to systematically summarize evidence on age-related changes in low-gamma (30–60 Hz) ASSRs measured with EEG or MEG across the human lifespan, integrating developmental, adult, and aging data to delineate how neural synchronization to external stimulation evolves from early childhood to older adulthood.

## 2 Methods

### 2.1 Review design and objectives

This study was conducted in line with the Preferred Reporting Items for Systematic reviews and Meta-Analyses extension for Scoping Reviews (PRISMA-ScR) (Tricco et al., 2018). The protocol was not prospectively registered. The aim was to summarize age-related differences in ASSRs recorded with EEG or MEG across the human lifespan. The research question was defined using the PICOS framework: Population (P) - humans across the lifespan, including typically developing and clinical groups; Intervention/Exposure (I) - EEG or MEG measurement of ASSRs elicited by periodic auditory stimulation; Comparison (C) - between-age group comparisons or age as a continuous predictor; Outcomes (O) - quantitative ASSR measures (amplitude, inter-trial phase coherence, phase-locking factor, power, signal-to-noise ratio, latency, or topographic features, etc.); Study design (S) - peer-reviewed human studies with cross-sectional or longitudinal designs.

### 2.2 Search strategy

The literature search aimed to identify all peer-reviewed human studies examining age-related effects on ASSRs measured with EEG or MEG. Two databases, PubMed and Scopus, were searched to ensure comprehensive coverage across biomedical and interdisciplinary sources. No date restrictions were applied, and only English-language full-text publications were considered. The search targeted three conceptual domains: (i) developmental stages (childhood and adolescence), (ii) aging and older adulthood, and (iii) general age or maturation effects across the lifespan. Each domain was represented by a dedicated Boolean query, detailed below.

### 2.3 Search procedure

The search was conducted systematically in accordance with the predefined strategy. Both PubMed and Scopus databases were queried, with no temporal limitations. Three complementary searches were performed in each database, targeting studies on childhood/adolescence, aging, and general maturation.

The search strategy combined terminology related to ASSRs (*auditory steady-state response, steady-state auditory evoked potential, ASSR, SSAEP*) with population-specific terms for developmental stages (*child, infant, adolescent, teen, pediatric*) and aging (*aging, older adults, elderly, senescence*). It also included broader age-related concepts (*age-related, maturation, lifespan, development, age effects*). Finally, all searches constrained results to studies using EEG or MEG methods (*EEG, electroencephalography, MEG, magnetoencephalography*). Complete search formulations are provided in Supplementary material.

### 2.4 Eligibility criteria

Studies were included if they involved human participants of any age, used EEG or MEG to record ASSRs elicited by periodic amplitude- or frequency-modulated sounds within the gamma range (typically 30–80 Hz), and reported age-related analyses through group comparisons or correlations. Eligible studies provided quantitative electrophysiological measures and were not limited to hearing-threshold precision estimation. Only original, peer-reviewed articles published in English were included.

Exclusion criteria encompassed non-human or invasive studies, the absence of age-related data, and investigations limited to behavioral or hemodynamic measures. Reviews, case reports, editorials, and conference abstracts, as well as duplicates and non-English full texts, were excluded.

### 2.5 Study selection process

**Fig. 1:**
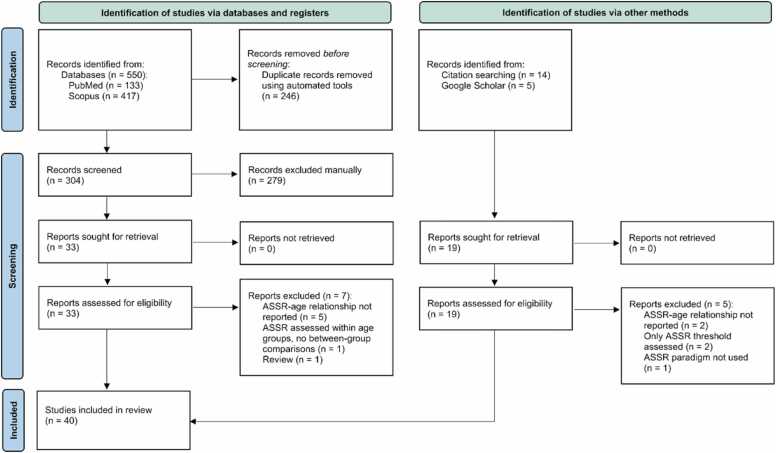
PRISMA diagram representing the article inclusion process.

All search results were imported into Rayyan (rayyan.ai, Ouzzani et al., 2016) for reference management and screening, resulting in a total of 550 records. Automatic and manual de-duplication were performed prior to screening. Three reviewers independently assessed titles and abstracts against the eligibility criteria, resolving disagreements through discussion or, when necessary, consultation with a fourth reviewer. After removing 246 duplicates, 304 unique records were screened, of which 26 studies met all inclusion criteria and were retained for qualitative synthesis. Subsequently, the reference lists of the retained articles were screened to identify relevant studies. Additionally, a nonsystematic search was conducted in Google Scholar to identify any potentially missed articles. The additional searches resulted in the inclusion of 14 more papers in this review. The full selection process is summarized in the PRISMA flow diagram (Fig. 1).

### 2.6 Data extraction

For each included study, relevant data were extracted into a structured spreadsheet. Extracted information included study identifiers (authors, year, DOI), participant characteristics (sample size, age range, sex distribution, and clinical status), and details of auditory stimulation and recording. Stimulation parameters comprised modulation type and frequency, carrier signal, intensity, duration, and presentation mode. Recording information specified whether EEG or MEG was used, the number of channels, and whether analyses were performed at the sensor or source level. Reported ASSR outcomes (such as amplitude, power, inter-trial phase coherence (ITPC), phase-locking factor (PLF), signal-to-noise ratio (SNR), and latency) were recorded along with the direction and significance of any age-related effects and hemispheric asymmetries. Results were extracted with a focus on responses in the 30–60 Hz frequency range (for single frequency stimulation) or at peak gamma frequency (for sweep-like stimulation). Data extraction was conducted independently by two reviewers, and discrepancies were resolved through verification and consensus.

## 3 Results

A formal quality appraisal of the included studies was not conducted, as the review aimed to map the existing evidence rather than evaluate study bias. The findings were integrated through qualitative narrative synthesis. Data were thematically organized according to developmental stage (childhood/adolescence, adulthood, aging) and clinical status, as well as by methodological factors such as recording modality, stimulus type, and modulation frequency. The extracted information is provided in Table 1.

**Table 1:** Available information extracted from the included studies regarding samples (sample and group types and sizes, sex composition, mena age, standard deviation and ranges), auditory stimulation (stimulation frequency and carrier, stimulus and inter-stimulus-interval duration, number of repetitions, monaural/binaural stimulation, intensity and instruction during the stimulation), data acquisition (EEG/MEG and the number of electrodes/channels), analysis (measure and ROI) and results (ASSR relationship with age or between-group comparisons).

	Study	Sample	Stimulus	Stimulation	Recording	Analysis	Age-related findings
1	Adalilar et al. (2025)	Healthy subjects Group 1 (N = 18) Age: 20–26 years Group 2 (N = 10) Age: 6–10 years Group 3 (N = 7) Age: 11–14 years	37, 40, 43, 77, 80, 83 Hz AM white noise carrier	Stimulus condition (6 frequencies x left/right/binaural) lasted 307.2 s Monaurally and binaurally 70 dB SPL Silent movie	EEG, 64 electrodes	Amplitude, SNR, phase lag, lateralization Source level: TP7, CP5, P9, P5, P7, PO7, PO3, O1, TP8, CP6, P10, P6, P8, PO8, PO4, and O2	Age was positively related to 40-Hz ASSR amplitude (only right ear stimulation), SNR and latency
2	Ahlfors et al. (2024)	Subjects with ASD (N = 22, 4 F) Age: 13.6 ± 0.6 (7−17) years Healthy subjects (N = 31,4 F) Age: 13.1 ± 0.6 (6−17) years	25 and 43 Hz AMb roadband noise carrier	1200 ms duration (300–400 ms ISI) 100 repetitions Binaurally 65 dB SPL	MEG, 306 channels	ITPC Source level: bilateral auditory cortex	Age positively predicted 43-Hz ASSR ITPC
3	Aoyagi et al. (1994)	Healthy subjects Group 1 (N = 5) Age: 8.6 (4−15) months Group 2 (N = 5) Age: 3 years 4 months (2 years 3 months – 4 years 11 months) Group 3 (N = 10) Age: 11 years 4 months (9 years 1 month – 15 years 6 months) Group 4 (N = 10) Age: 31.4 (22−46) years	20–200 Hz (20 Hz steps) AM 1000-Hz carrier	Monaurally 50 dB nHL During sleep	EEG, 1 electrode	Amplitude, SNR and ITPC Sensor level: vertex	40-Hz ASSR ITPC and SNR was higher in adults and older children compared to younger children
4	Arutiunian et al. (2022)	Healthy subjects (N = 30, 12 F) Age: 9.1 ± 1.5 (7.06–12.03) years	40 Hz AM 1000 Hz carrier	1000 ms duration (2000 ms ISI) 90 repetitions Binaurally 83.7 dB SPL Fixation cross	MEG, 306 channels	ITPC and amplitude Source level: bilateral temporal cortices	40-Hz ASSR ITPC correlated positively, while amplitude correlated negatively with age, only in the right hemisphere
5	Arutiunian et al. (2023)	Subjects with ASD (N = 20, 5 F) Age: 10.03 ± 1.7 (8.02–14.01) years Healthy subjects (N = 20, 9 F) Age: 9.11 ± 1.3 (7.02–12.03) years	40 Hz AM 1000 Hz carrier	1000 ms duration (2000 ms ISI) 90 repetitions Binaurally 83.7 dB SPL Fixation cross	MEG, 306 channels	ITPC Source level: bilateral auditory cortex	40-Hz ASSR ITPC correlated positively with age in both hemispheres in healthy group only
6	Cho et al. (2015)	Healthy subjects (N = 181, 89 F) Age: 14.5 ± 4.4 (8−22) years Group 1 (N = 44, 21 F) Age: 8–10 years Group 2 (N = 32, 16 F) Age: 11–13 years Group 3 (N = 37, 19 F) Age: 14–16 years Group 4 (N = 36, 18 F) Age: 17–19 years Group 5 (N = 32, 15 F) Age: 20–22 years	20, 30, 40 Hz click trains	500 ms duration Binaurally 65 ± 5 dB Oddball paradigm	EEG, 128 electrodes	Amplitude and ITPC Sensor level: 14 electrodes centered at FCz	40-Hz ASSR amplitude and ITPC linearly increased from age group 1–3, but decreased from age group 3–5. No significant differences in 30-Hz ASSR measures
7	Darrell et al. (2025)	Subjects with ASD (N = 53, 10 F) Age: 10.84 ± 1.5 years Healthy subjects (N = 35, 18 F) Age: 10.4 ± 1.8 years	27, 40 Hz click trains	500 ms duration (488–788 ms ISI) 200 repetitions Binaurally 60 dB SPL Oddball paradigm	EEG, 64 electrodes	Power Sensor level: FC3, FCz, FC4	40-Hz ASSR power correlated positively with age in a combined and ASD group
8	Dimitrijevic et al. (2004)	Hearing-impaired subjects (N = 10, 6 F) Age: 75 (57−86) years Healthy subjects Group 1 (N = 10, 5 F) Age: 28 (22−33) years Group 2 (N = 10, 6 F) Age: 68 (60−82), years	39–55 and 78–100 Hz AM and FM 500, 1000, 1500 and 2000 Hz carrier	Binaurally 70 dB SPL During sleep	EEG, 1 electrode	Amplitude Sensor level: vertex	No significant differences in ASSR amplitudes between normal-hearing age groups
9	Dobri et al. (2023)	Healthy subjects Group 1 (N = 19, 11 F) Age: 23.8 ± 6.2 (19−28) years Group 2 (N = 19, 12 F) Age: 76.1 ± 6.2 (69−87) years	40 Hz AM 400 Hz carrier	2050 ms total duration, consisting of 400-ms bursts separated by 12.5 ms pauses (1950-ms ISI) Presented alone combined with babble noise 300 repetitions per condition Binaurally 60 dB above individual threshold Passive listening	MEG, 151 channels	Amplitude Source level: bilateral auditory cortices	Significantly higher 40-Hz ASSR amplitude in older vs young group only in noise condition. Significant positive correlation between 40-Hz ASSR amplitude with age in older group
10	Edgar et al. (2016)	Subjects with ASD (N = 55, 4 F) Age: 10.6 ± 1.5 (8−14) years Healthy subjects (N = 56, 3 F) Age: 10.1 ± 1.5 (7−14) years	40 Hz AM 500 Hz carrier	1 s duration (4 s ISI) Binaurally 45 dB above hearing threshold Silent movie	MEG, 306 channels	Power and ITPC Source level: bilateral auditory cortex	Age was a positive predictor of 40-Hz ASSR power (right hemisphere) and ITPC (both hemispheres)
11	Edgar et al. (2017)	Healthy subjects (N = 53, 18 F) Age: 39.6 ± 12.1 years	40 Hz AM 500 Hz carrier	1 s duration (4 s ISI) 88 ± 18 average repetitions Binaurally 35 dB above individual hearing threshold Fixation cross	MEG, 306 channels EEG, 60 electrodes	Power and ITPC Source level: bilateral auditory cortex Sensor level: Cz, Fz and 18 electrodes per hemisphere	Significant negative correlation of age with 40-Hz ASSR ITPC and power in the left hemisphere at source level
12	Edgar et al. (2018)	Subjects with SZ (N = 41, 7 F) Age: 40.3 ± 11.6 (20–60) years Healthy subjects (N = 55, 19 F) Age: 39.6 ± 11.9 (21–58) years	40 Hz AM 500 Hz carrier	1 s duration (4 s ISI) 87 ± 18 (SZ) or 92 ± 22 (healthy) average repetitions Binaurally 35 dB above individual hearing threshold	MEG, 306 channels EEG, 60 electrodes	Power and ITPC Source level: Bilateral temporal cortices	Significant negative correlation of age with 40-Hz ASSR ITPC and power in healthy controls but not SZ patients
13	Farahani et al., 2020b, Farahani et al., 2020a	Healthy participants Group 1 (N = 19, 10 F) Age: 20–30 years Group 2 (N = 20, 10 F) Age: 50–60 years Group 3 (N = 16, 11 F) Age: 70–80 years	4, 2, 40 and 80 Hz AM white noise carrier	300 s duration Monaurally 70 dB SPL Silent video	EEG, 64 electrodes	Amplitude and ITPC Source level: multiple cortical and subcortical ROIs	40-Hz ASSR amplitude was higher in multiple ROIs in older compared to younger and middle-aged groups; higher in younger compared to middle-aged group; 40-Hz ASSR ITPC was higher in frontal cortical ROIs in older compared to younger and middle-aged group
14	Goossens et al. (2016)	Healthy subjects Group 1 (N = 19, 10 F) Age: 22 ± 1 years Group 2 (N = 20, 10 F) Age: 52 ± 2 years Group 3 (N = 14, 10 F) Age: 74 ± 3 years	4, 20, 40, 80 Hz AM white noise carrier	300 s duration Monaurally and binaurally 70 dB SPL Silent movie	EEG, 64 electrodes	SNR, sensor level: TP7/8, CP5/6, P9/10, P7/8, P5/6, PO7/8, PO3/4, O1/2	No differences in 40-Hz ASSR SNR between age groups
15	Griskova-Bulanova et al. (2013)	Healthy subjects (N = 46, all M) Group 1 (N = 13) Age: 20–30 years Group 2 (N = 13) Age: 30–40 years Group 3 (N = 9) Age: 40–50 years Group 4 (N = 11) Age: 50–60 years	40 Hz click trains	500 ms duration (1–1.5 s ISI) 60 repetitions Binaurally 60 dB SPL Fixation cross with passive listening	EEG, 9 electrodes	Amplitude and ITPC Sensor level: F3, Fz, F4, C3, Cz, C4, P3, Pz, P4	Age negatively correlated to 40-Hz ASSR ITPC and amplitude
16	Griskova-Bulanova et al. (2020)	Subjects with SZ (N = 18, all M) Age: 38 ± 14 years Healthy subjects (N = 18, all M) Age: 42 ± 13 years	1–120 Hz sweeps AM 440 Hz carrier	500 ms duration (700–1000 ms ISI) 450 repetitions Binaurally 60 dBA Silent movie	EEG, 9 electrodes	Amplitude and ITPC Sensor level: Fz and Cz	No correlation of ASSR with age
17	Herdman (2011)	Healthy subjects Group 1 (N = 12, 6 F) Age: 12 ± 0.7 years Group 2 (N = 13, 7 F) Age: 22 ± 2.3 years	40 Hz AM 800 or 1200 Hz carrier	500 or 175 ms duration (1000–1500 ms ISI) 96 or 384 repetitions Binaurally 70 dB SPL Oddball paradigm	MEG, 151 channels	Amplitude Source level: bilateral auditory cortex	Significantly higher 40-Hz ASSR amplitude in adults compared to children
18	Irazabal et al. (2024)	Healthy subjects (N = 23, 14 F) Age: 115.26 ± 57.65 months (1–18 years)	1–120 Hz sweeps AM 1200 Hz carrier	1600 ms duration (400 ms ISI) 500 repetitions Binaurally 85 dB SPL Silent video	EEG, 19 electrodes	Evoked power and ITPC Sensor-level: F3, Fz, F4	Significant positive correlation between ITPC at peak low gamma frequency and age; no correlation between evoked power and age
19	Johnson et al. (1988)	Healthy subjects Group 1 (N = 5, all F) Age: 38 (36−40) years Group 2 (N = 7, all F) Age: 69.6 (65−77) years	40 Hz trains of 1000 Hz bursts	500 repetitions Binaurally Eyes-closed passive listening	EEG, 21 electrodes	Amplitude Sensor level: whole-head	No difference between age groups in 40-Hz ASSR
20	Kim et al. (2019)	Subjects with SZ (N = 33, 17 F) Age: 42.21 ± 10.99 (21−60) years Healthy subjects (N = 30, 17 F) Age: 43.33 ± 12.95 (23–64) years	40 Hz click trains	500 ms duration (3050–3500 ISI) 150 repetitions Binaurally 80 dB SPL Fixation cross with active listening	EEG, 64 electrodes	Power and ITPC Sensor level: Cz	No significant correlation between age and 40-Hz ASSR
21	Larsen et al. (2018)	Subjects with 22q11.2 deletion syndrome (N = 18, 5 F) Age: 15.39 ± 2.45 years Healthy subjects (N = 27, 9 F) Age: 15.96 ± 2.71 years	40 Hz click trains	1 s duration (2 s ISI) 85 dB SPL 120 repetitions Binaurally Fixation cross with passive listening	EEG, 128 electrodes	Power and ITPC Sensor level: Cz	Age negatively predicted 40-Hz ASSR power and ITPC in combined 22q11.2 and control group
22	Leigh-Paffenroth and Fowler (2006)	Healthy subjects Group 1 (N = 16, 12 F) Age: 29 years Group 2 (N = 12, 6 F) Age: 69.9 years	20, 40 or 90-Hz AM 500 Hz or 1000 Hz carrier	Monaurally	EEG, 1 electrode	ITPC Sensor level: Fz	40-Hz ASSR ITPC was significantly higher in younger subjects compared to older only at 500 Hz carrier frequency
23	Manasevich et al. (2025)	Healthy subjects (N = 57, 27 F) Age: 64.35 ± 11.96 (42−82) months	40 Hz click trains	500 ms duration (500–800 ms ISI) Binaurally 65 dB SPL Silent video	EEG, 32 electrodes	ITPC Source level: Fz	Significant positive correlation of 40-Hz ASSR with age
24	Mancini et al. (2022)	Subjects with 22q11.2 deletion syndrome (N = 58, 26 F) Age: 17.6 ± 6.9 years Healthy subjects (N = 48, 24 F) Age: 17.7 ± 6.2 years Both groups divided into age bins: 1) 7–13 years 2) 14–18 years 3) ≥ 19 years	40 Hz AM 1000-Hz carrier	2 s duration (1.5–2.5 s ISI) 100 repetitions Binaurally Oddball paradigm	EEG, 256 electrodes	Power and ITPC Sensor level: cluster of electrode around FCz	Significant linear increase in 40-Hz ASSR power and ITPC from childhood to adulthood in control group only
25	Maurizi et al. (1990)	Healthy subjects Group 1 (N = 32, 16 F) Age: 1–3 days Group 2 (N = 10, 3 F) Age: 5–8 years	40 Hz trains of 500 Hz bursts	1000 bursts Monaurally Decreasing intensity from 70 dB nHL to threshold During sleep (neonates) or passive listening (children)	EEG, 1 electrode	Amplitude, test-retest reliability Sensor level: upper forehead	Increased 40-Hz ASSR amplitude and test-retest reliability in children as compared to neonates
26	McKeon et al. (2024)	Healthy subjects (N = 164, F = 87) Age: 10–32 years	20, 30, 40 Hz click trains	500 ms duration (605 ms ISI) 150 repetitions Binaurally Passive listening	EEG, 64 electrodes	Evoked power, spontaneous power and SNR Sensor level: F3, F5, F7, F1, F2, F4, F6, F8, AFz, AF1, AF2, Fp1, Fp2, Fz, AF5, AF6	ASSR SNR increased through adolescence only; no change in evoked power with age
27	Neklyudova et al. (2021)	Healthy subjects Group 1 (N = 13, 7 F) Age: 16.04 ± 1.9 (12−18) years Group 2 (N = 19, 14 F) Age: 7.8 ± 2.6 (3−12) years	40 Hz click tains	500 ms duration (500–800 ms ISI) 150 repetitions Binaurally 80 dB SPL Silent video	EEG 32 electrodes	Amplitude Sensor level: Fz	40-Hz ASSR amplitude was significantly higher in “old” group compared to “young” group
28	Neklyudova et al. (2024)	Subjects with RTT (N = 43, all F) Age: 8.19 ± 3.84 (2.92–17.1) years Healthy subjects (N = 43, 26 F) Age: 8.27 ± 3.87 (2.58–17.45) years	40 Hz click trains	500 ms duration (500–800 ms ISI) 150 repetitions Binaurally 65 dB SPL Silent video	EEG, 28 electrodes	Amplitude Sensor level: FCz	Significant positive correlation between age and 40-Hz ASSR amplitude only in healthy group
29	Ono et al. (2020)	Subjects with ASD (N = 23, 5 F) Age: 74.8 ± 11.2 months Healthy subjects (N = 32, 12 F) Age: 69.7 ± 6.2 months	20, 40 Hz AM 1000 Hz carrier	1 s duration (900–1100 ms ISI) Binaurally 70 dB SPL Silent video	MEG, 151 channels	Power and ITPC Source level: bilateral auditory cortex	Positive correlation of 40-Hz ASSR ITPC and age in the right hemisphere only in healthy group
30	Poulsen et al. (2007)	Healthy subjects (N = 23, 11 F) Age: 29 (19−45) years	40 Hz FM 1000 Hz carrier 10–100 Hz sweeps AM white noise carrier	FM stimulus: 1000 ms duration (1000 ± 100 ms ISI) 400 repetitions AM stimuli: 15.36 s up part, 15.36 s down part 10 repetitions 65 dB SPL (FM stimuli) and 55 dB SPL (AM stimuli) Binaurally Silent video	EEG, 128 electrodes	Amplitude Sensor level: Cz Source level: bilateral temporal cortex and brainstem	Positive relationship between 40-Hz ASSR amplitude (FM stimuli) and age, both in sensor and source level; No relationship between ASSR amplitude and age at peak frequency (AM stimuli)
31	Poulsen et al. (2009)	Healthy subjects Group 1 (N = 60, 28 F) Age (T1): 10 ± 0.39 (9.4 – 10.8) years Age (T2): 11.5 ± 0.39 (10.9 – 12.3) years Group 2 (N = 23, 11 F) Age: 29 (19−45) years	40 Hz FM 1000 Hz carrier 10–100 Hz sweeps AM white noise carrier	FM stimulus: 1000 ms duration (1000 ± 100 ms ISI) 400 repetitions AM stimuli: 15.36 s up part, 15.36 s down part 10 repetitions 65 dB SPL (FM stimuli) and 55 dB SPL (AM stimuli) Binaurally Silent video	EEG, 128 electrodes	Amplitude Sensor level: Cz Source level: bilateral temporal cortex and brainstem	Significantly higher 40-Hz ASSR in adults vs children; significant increase in amplitude at T2 compared to T1 in children group; no significant difference in EFR amplitude at the peak frequency between adult and children groups and within children group
32	Purcell et al. (2004)	Healthy subjects Group 1 (N = 25, 20 F) Age: 18–43 years Group 2 (N = 13, 6 F) Age: 60–78	20–600 Hz sweeps AM white noise carrier	30 s duration, 15-s up and 15-s down parts Monaurally 50 or 60 dB SPL Silent movie	EEG, 1 electrode	Amplitude, sensor level: Cz	No differences in ASSR amplitude at peak frequency between age groups
33	Roberts et al. (2021)	Subjects with ASD (N = 80, 12 F) Age: 11.72 ± 0.26 years Healthy subjects (N = 40, 6 F) Age: 11.94 ± 0.44 years	10–100 Hz sweep AM 500 Hz carrier	30 s sweeps of 15-s up and 15-s down parts (9 s ISI) 20 repetitions Binaurally 45 dB SL	MEG, 275 channels	ITPC Source level: bilateral auditory cortex	Age positively predicted ASSR ITPC at the peak frequency
34	Rojas et al. (2006)	Healthy subjects (N = 69, 32 F) Age: 25.62 ± 13.05 (5−52) years	40 Hz click trains	500 ms duration (1.5 s ISI) 150 repetitions Binaurally 65 dB SPL Silent video	MEG, 37 channels	Power Sensor level: channel with maximal power	Exponential relationship between 40-Hz ASSR and age, with sharp increase in early age and plateau in adulthood
35	Ross (2018)	Healthy participants Group 1 (N = 19, 12 F) Age: 22.4 (18−30) years Group 2 (N = 21, 14 F) Age: 70.9 (63−77) years	40 Hz AM 400, 800, 1200, 1600 and 2400 Hz carrier	13.6 s duration (16 s ISI) 22 repetitions Binaurally 60 dB SPL Fixation cross	MEG, 151 channels	Amplitude Source level: bilateral auditory cortex	Age positively predicted 40-Hz ASSR amplitude
36	Stapells et al. (1988)	Healthy subjects Group 1 (N = 18) Age: 42.1 ± 32.4 weeks (3 weeks – 28 months) Group 2 (N = 8) Age: 26–34 years	9–59 Hz (5 Hz steps) trains of 1000 Hz bursts or 43.4 Hz click trains	4096 repetitions of 1000-Hz bursts or 8192 repetitions of clicks Monaurally 70 dB nHL During sleep	EEG, 5 electrodes	Amplitude, phase Sensor level: Cz	ASSR around 40 Hz was significantly higher in adults compared to children
37	Stroganova et al. (2020)	Subjects with ASD (N = 35, all M) Age: 9.69 ± 1.5 (7.2–12.3) years Healthy subjects (N = 35, all M) Age: 10.08 ± 1.5 (7.3–12.9) years	40 Hz click trains	500 ms duration (1000 ms ISI) 100 repetitions Monaurally 60 dB SPL Silent video	MEG, 306 channels	ITPC Source level: bilateral auditory cortex	40-Hz ASSR ITPC correlated positively with age in both ASD and controls
38	Tang et al. (2016)	Healthy subjects Group 1 (N = 12, 4 F) Age: 49.3 months (3–5 years) Group 2 (N = 12, 7 F) Age: 28.8 (22−36) years	1–80 Hz sweep AM white noise carrier	9 s duration (900–1000 ms ISI) 100 (children) or 200 (adults) repetitions Binaurally 75 dB SPL Silent movie	MEG, 64 (children) and 160 (adults) channels	ITPC Source level: bilateral auditory cortex	ASSR ITPC was higher in adults compared to children at 15–80 Hz
39	Tlumak et al. (2015)	Healthy participants Group 1 (N = 16) Age: 29.69 ± 4.76 (20−39) years Group 2 (N = 16) Age: 51.94 ± 5.27 (40−59) years Group 3 (N = 16) Age: 63.37 ± 3.9 (60−79)	0.75, 1.25, 2.5, 5, 10, 20, 40 Hz AM 1000 Hz carrier	Right ear 70 dB SPL	EEG, 2 electrodes	Amplitude Sensor level: Cz	No differences in 40-Hz ASSR amplitude among age groups
40	Usui et al. (2023)	Healthy subjects (N = 67, 33 F) Age (T1): 13.4 ± 0.5 (12.3–14.3) years Age (T2): 16.1 ± 0.8 (14.4–18.2) years	20, 40 Hz click trains	500 ms duration (500 ms ISI) 200 repetitions Binaurally 80 dB Passive listening with eyes open	EEG, 2 electrodes	Power and ITPC Sensor level: Fz and Cz	No significant differences in 40-Hz ASSR power and ITPC between T1 and T2 measurements

**Fig. 2:**
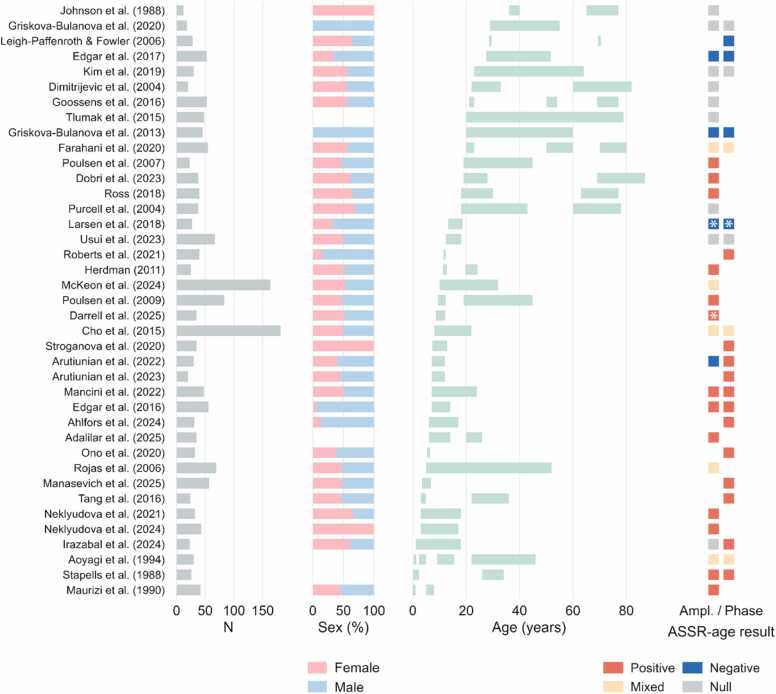
Summary of study characteristics: from the left, total sample size, sex distribution, age span and reported ASSR-age relationship. Only healthy cohorts are shown. Age spans were taken directly from reported minimum–maximum values when available or estimated as mean ± SD. ASSR-age relationships are shown for amplitude (left) and phase-based (right) measures separately. Red (positive) and blue (negative) represent ASSR increase and decrease with age (based either on correlations or between-group comparisons), respectively. Beige (mixed category) refers to studies showing non-linear relationships or variable results when comparing multiple age groups. Studies reporting null relationships between ASSR and age are labeled in grey (null). White asterisk marks the studies that assessed ASSR-age relationship in a combined sample of patients and controls. Edgar et al. (2018) was not included in the plot due to largely overlapping healthy participant sample with a previous study (Edgar et al., 2017). For more details, refer to Results section and Table 1.

Of the 40 studies included in this review, 3 investigated infant samples below one year of age (Aoyagi et al., 1994, Maurizi et al., 1990, Stapells et al., 1988), demonstrating that measurable 40-Hz ASSRs can already be detected in early infancy. Eighteen studies examined ASSR in early life, covering ages up to mid 20 s (Adalilar et al., 2025, Ahlfors et al., 2024, Arutiunian et al., 2022, Arutiunian et al., 2023, Cho et al., 2015, Darrell et al., 2025; Edgar et al., 2016; Herdman et al., 2011; Irazabal et al., 2024; Larsen et al., 2018; Manasevich et al., 2025; Mancini et al., 2022; Maurizi et al., 1990; Neklyudova et al., 2021, Neklyudova et al., 2024; Ono et al., 2020; Roberts et al., 2021; Stroganova et al., 2020), predominantly highlighting developmental increases in low-gamma ASSR. Ten studies primarily addressed adulthood and aging, including those that compared young, middle-aged, and older adult groups (Dimitrijevic et al., 2004, Dobri et al., 2023, Farahani et al., 2020b, Goossens et al., 2016; Griskova-Bulanova et al., 2013; Johnson et al., 1988; Leigh-Paffenroth and Fowler, 2006; Purcell et al., 2004; Ross, 2018; Tlumak et al., 2015), and reflecting non-uniform ageing effects, with 40-Hz synchronization ranging from diminished to preserved or even increased. Three studies employed a longitudinal design (McKeon et al., 2024, Poulsen et al., 2009, Usui et al., 2023), showing mixed findings in adolescent and adult participants. The remaining works involved adult cross-sectional or broad lifespan samples, ranging from early childhood or adolescence into late adulthood (Aoyagi et al., 1994, Edgar et al., 2017, Edgar et al., 2018, Griskova-Bulanova et al., 2020, Kim et al., 2019, Poulsen et al., 2007, Rojas et al., 2006, Stapells et al., 1988, Tang et al., 2016), further contributing to the overall picture of nonlinear developmental and aging trajectories. Sample characteristics and reported ASSR-age relationships are summarized in Fig. 2. Only data from healthy participants were included in this visual synthesis.

Additionally, 11 studies included clinical or neurodivergent populations alongside healthy controls. These comprised individuals with autism spectrum disorder (Ahlfors et al., 2024, Arutiunian et al., 2023, Darrell et al., 2025, Edgar et al., 2016, Stroganova et al., 2020), Rett syndrome (Neklyudova et al., 2024), 22q11.2 deletion syndrome (Larsen et al., 2018, Mancini et al., 2022), and schizophrenia-spectrum disorders (Edgar et al., 2018, Griskova-Bulanova et al., 2020, Kim et al., 2019).

Across the reviewed studies, 18 reported positive associations between age and ASSR measures, i.e., increased amplitude and/or phase-locking with age (Adalilar et al., 2025, Ahlfors et al., 2024, Arutiunian et al., 2023, Darrell et al., 2025, Dobri et al., 2023, Edgar et al., 2016, Herdman, 2011, Manasevich et al., 2025, Mancini et al., 2022, Maurizi et al., 1990, Neklyudova et al., 2021, Neklyudova et al., 2024, Ono et al., 2020, Roberts et al., 2021, Ross, 2018, Stapells et al., 1988, Stroganova et al., 2020, Tang et al., 2016), 5 reported negative associations (Edgar et al., 2017, Edgar et al., 2018; Griskova-Bulanova et al., 2013; Larsen et al., 2018; Leigh-Paffenroth and Fowler, 2006), and 8 found no significant age effects (Dimitrijevic et al., 2004, Goossens et al., 2016, Griskova-Bulanova et al., 2020, Johnson et al., 1988, Kim et al., 2019, Purcell et al., 2004, Tlumak et al., 2015, Usui et al., 2023). Other studies described mixed findings, including different results for amplitude and frequency-based measures (Arutiunian et al., 2022, Irazabal et al., 2024), non-linear ASSR trajectories with age (McKeon et al., 2024, Rojas et al., 2006) or divergent ASSR changes depending on age groups (Aoyagi et al., 1994, Cho et al., 2015, Farahani et al., 2020b).

ASSRs were most frequently elicited by 40-Hz amplitude-modulated (AM) tones (Adalilar et al., 2025, Ahlfors et al., 2024, Aoyagi et al., 1994, Arutiunian et al., 2022, Arutiunian et al., 2023, Dobri et al., 2023, Edgar et al., 2016, Edgar et al., 2017, Edgar et al., 2018, Farahani et al., 2020b, Goossens et al., 2016, Herdman, 2011, Leigh-Paffenroth and Fowler, 2006, Mancini et al., 2022, Ono et al., 2020, Ross, 2018, Tlumak et al., 2015) or click trains (Cho et al., 2015, Darrell et al., 2025; Griskova-Bulanova et al., 2013; Johnson et al., 1988; Kim et al., 2019; Larsen et al., 2018; Manasevich et al., 2025; Maurizi et al., 1990; McKeon et al., 2024; Neklyudova et al., 2021, Neklyudova et al., 2024; Rojas et al., 2006; Stapells et al., 1988; Stroganova et al., 2020; Usui et al., 2023). Several studies employed multi-frequency sweeps or chirp stimuli (Irazabal et al., 2024, Griskova-Bulanova et al., 2020, Purcell et al., 2004, Roberts et al., 2021, Tang et al., 2016), or compared AM and frequency-modulated (FM) tones to examine resonance profiles across the gamma range (Dimitrijevic et al., 2004, Poulsen et al., 2007, Poulsen et al., 2009).

EEG was used in 28 studies, either employing lower density setups of 1–28 electrodes (Aoyagi et al., 1994, Dimitrijevic et al., 2004, Irazabal et al., 2024, Griskova-Bulanova et al., 2020, Johnson et al., 1988, Leigh-Paffenroth and Fowler, 2006, Maurizi et al., 1990, Neklyudova et al., 2024, Purcell et al., 2004, Stapells et al., 1988, Tlumak et al., 2015) or higher density recordings with 32–256 channels (Adalilar et al., 2025, Cho et al., 2015, Darrell et al., 2025, Edgar et al., 2017, Edgar et al., 2018, Farahani et al., 2020b, Goossens et al., 2016, Kim et al., 2019, Larsen et al., 2018, Manasevich et al., 2025, Mancini et al., 2022, McKeon et al., 2024, Neklyudova et al., 2021, Poulsen et al., 2007, Poulsen et al., 2009, Usui et al., 2023), while MEG was applied in 14 studies (Ahlfors et al., 2024, Arutiunian et al., 2022, Arutiunian et al., 2023, Dobri et al., 2023, Edgar et al., 2016, Edgar et al., 2017, Edgar et al., 2018, Herdman, 2011, Ono et al., 2020, Roberts et al., 2021, Rojas et al., 2006, Ross, 2018, Stroganova et al., 2020, Tang et al., 2016), employing 151–306-channel whole-head systems.

At the analysis level, 26 studies reported sensor-space measures, typically at frontocentral electrodes such as Fz, FCz or Cz (Aoyagi et al., 1994, Cho et al., 2015, Darrell et al., 2025, Dimitrijevic et al., 2004, Edgar et al., 2017, Irazabal et al., 2024, Goossens et al., 2016, Griskova-Bulanova et al., 2020, Johnson et al., 1988, Kim et al., 2019, Larsen et al., 2018, Leigh-Paffenroth and Fowler, 2006, Manasevich et al., 2025, Mancini et al., 2022, Maurizi et al., 1990, McKeon et al., 2024, Neklyudova et al., 2021, Neklyudova et al., 2024, Poulsen et al., 2007, Poulsen et al., 2009, Purcell et al., 2004, Rojas et al., 2006, Stapells et al., 1988, Tlumak et al., 2015, Usui et al., 2023), but also temporal-parietal electrodes (Adalilar et al., 2025), while 16 conducted source-space analyses (Ahlfors et al., 2024, Arutiunian et al., 2022, Arutiunian et al., 2023, Dobri et al., 2023, Edgar et al., 2016, Edgar et al., 2017, Edgar et al., 2018, Farahani et al., 2020b, Herdman, 2011, Ono et al., 2020, Poulsen et al., 2007, Poulsen et al., 2009, Roberts et al., 2021, Ross, 2018, Stroganova et al., 2020, Tang et al., 2016). Nearly all MEG investigations localized activity to superior temporal regions and several demonstrated right-hemisphere dominance or asymmetric developmental trajectories (Ahlfors et al., 2024, Arutiunian et al., 2022, Edgar et al., 2016, Ono et al., 2020, Roberts et al., 2021).

## 4 Discussion

This review indicates that gamma-range ASSRs follow a broadly non-linear lifespan pattern, with robust developmental strengthening, relative stabilization in early adulthood, and heterogeneous changes in aging. However, coverage across the full lifespan remains uneven, with sparse data in early childhood, late adulthood, and longitudinal designs, limiting precise delineation of maturation and decline trajectories. Consequently, our understanding of how gamma-range ASSRs evolve across the lifespan remains incomplete and biased toward developmental stages, underscoring the need for harmonized longitudinal and lifespan-spanning investigations. To contextualize this uneven coverage, findings are summarized below across three main domains: developmental trajectory, adulthood and aging, and clinical or neurodivergent populations.

### 4.1 Developmental trajectory (childhood to early adulthood)

Developmental studies indicate that the strength of ASSRs increases markedly from infancy through adolescence. Evidence from fetal MEG further suggests that the neural capacity for rhythmic auditory synchronization begins to emerge before birth (Niepel et al., 2020): steady-state responses were detected in fetuses between 30 and 38 weeks of gestation at 27 Hz, whereas responses around 42 Hz were absent, indicating that higher-frequency gamma synchronization develops only later in postnatal life.

The earliest post-natal investigations, including infant cohorts (Aoyagi et al., 1994, Maurizi et al., 1990, Stapells et al., 1988) demonstrated weak or unreliable 40-Hz responses during the first months of life, followed by progressive strengthening across early childhood. Subsequent studies of typically developing children and adolescents (e.g., Ahlfors et al., 2024; Edgar et al., 2016) reported positive correlations between age and response magnitude or phase-locking, frequently with right-hemisphere predominance (e.g., Arutiunian et al., 2022; Ono et al., 2020). Group-based comparisons further demonstrated higher amplitudes in adolescents or adults relative to younger children (e.g., Herdman, 2011; Neklyudova et al., 2021), reinforcing a picture of gradual refinement of neural synchrony during early development. Longitudinal data also support this trajectory: in a repeated-measures design, Usui et al. (2023) found stable 40-Hz responses between mid-adolescent assessments, suggesting that the major phase of ASSR maturation likely concludes by early adolescence.

Similar developmental increases in gamma activity have been observed in resting-state and task-based EEG/MEG studies, consistent with progressive maturation of inhibitory circuitry and excitation–inhibition balance (Candelaria-Cook et al., 2022, McKeon et al., 2023) and reflecting progressive stabilization, inhibitory refinement, and efficiency of cortical networks (Rhodes et al., 2025). These developmental changes have been attributed to the maturation of inhibitory (GABAergic) circuitry and refinement of excitation–inhibition balance (Gogolla et al., 2009, Kilb, 2012, Uhlhaas and Singer, 2010), as well as structural processes such as myelination and synaptic pruning that enhance long-range synchrony and network efficiency (Fair et al., 2009, Giedd et al., 2008, Whitford et al., 2007).

Collectively, these findings delineate a developmental trajectory that begins with weak or inconsistent gamma synchronization in infancy, progresses through childhood and adolescence, and stabilizes in early adulthood as auditory cortical circuits reach functional maturity, providing a continuous framework for interpreting the lifespan changes described below.

### 4.2 Aging trajectory (adulthood and aging)

In adulthood and aging, the trajectory of gamma-range ASSRs becomes increasingly heterogeneous. Several studies reported attenuated 40-Hz responses with advancing age (e.g., Griskova-Bulanova et al., 2013; Kim et al., 2019). Conversely, other investigations demonstrated enhanced amplitude or phase-locking in older adults (e.g., Dobri et al., 2023; Ross, 2018). The majority of studies found no significant age-related differences in 40-Hz responses (e.g., Goossens et al., 2016; Purcell et al., 2004).

Comparable age-related patterns have been observed across broader indices of gamma-band activity, with resting-state or task-based EEG/MEG studies showing that gamma power and peak frequency tend to decline with advancing age (Güntekin et al., 2023, Murty et al., 2020, Park et al., 2022). These findings are consistent with age-related alterations in inhibitory tone and cortical synchronization (Heise et al., 2022, Porges et al., 2021).

Beyond neurochemical and network-level factors, peripheral and subcortical auditory changes also contribute to age-related variability in ASSRs. Cochlear synaptopathy and brainstem dysfunction reduce hearing sensitivity and temporal precision, weakening early auditory coding (Ouda et al., 2015, Parthasarathy and Kujawa, 2018, Sergeyenko et al., 2013). Notably, the integrity of both peripheral hearing and cortical structure has been identified as essential for robust ASSRs, with smaller auditory cortical volumes and compromised white-matter connectivity predicting weaker gamma synchronization (Kim et al., 2019, Koshiyama et al., 2024, Schuler et al., 2022). Neuroimaging evidence further demonstrates diminished auditory cortical activation and reorganization of tonotopic maps in aging (Profant et al., 2015), alongside gray- and white-matter alterations in auditory and association regions reflecting large-scale cortical remodeling across the lifespan (de Mooij et al., 2018). These sensory and structural factors, together with neurochemical decline, likely interact with cortical E/I dynamics, amplifying the interindividual variability observed in aging cohorts.

Collectively, these findings suggest that, rather than a uniform decline, the adult–elderly transition in gamma synchronization is characterized by greater interindividual variability and regionally differentiated modulation of cortical oscillations. This variability likely reflects the combined influence of neurochemical decline, compensatory network recruitment, and methodological diversity across studies, underscoring that age-related changes in ASSRs are not purely degenerative but represent a complex reorganization of auditory cortical dynamics with aging.

### 4.3 Clinical populations and atypical development

Studies involving clinical or neurodivergent populations provide additional insight into how the normative age-related trajectory of 40-Hz ASSRs may be altered by neurodevelopmental or neuropsychiatric conditions affecting cortical synchronization.

In autism spectrum disorder (ASD), several studies have reported positive correlations between age and 40-Hz ASSR power or phase-locking (e.g., Arutiunian et al., 2023; Darrell et al., 2025). However, these associations are typically weaker, delayed, or lateralized compared with typically developing controls (Ono et al., 2020; Ahlfors et al., 2024). In studies that included 22q11.2 deletion syndrome (Mancini et al., 2022) and Rett syndrome (Neklyudova et al., 2024) patients, positive age–ASSR relationships were observed only in healthy participants. Although the available evidence remains limited, these findings suggest that the typical age-related strengthening of gamma synchronization may be delayed or dysregulated in neurodevelopmental conditions, reflecting atypical maturation and E/I balance within auditory cortical networks. This interpretation is further supported by recent animal-model findings: in Fragile X and PTEN-deletion mouse models, the maturation of 40-Hz temporal processing is delayed or dysregulated, showing region- and sex-specific differences (Croom et al., 2023, Croom et al., 2024, Croom et al., 2024).

Similarly, in schizophrenia-spectrum disorders, Kim et al. (2019), Griskova-Bulanova et al. (2020), and Edgar et al. (2018) reported no significant associations between age and ASSR measures. Meta-analytic evidence supports this interpretation, showing reductions in 40-Hz ASSR amplitude and phase-locking with only weak age dependence, manifesting as slightly larger deficits in younger patients and a developmentally anchored disruption of gamma synchronization (Thuné et al., 2016, Zouaoui et al., 2023).

Taken together, findings across clinical groups demonstrate that disturbances in NMDA-receptor-mediated E/I balance can delay, attenuate, or uncouple the typical age-related evolution of the 40-Hz ASSR. These deviations from the normative trajectory provide a sensitive window into atypical neurodevelopment and the pathophysiology of altered cortical synchronization across neuropsychiatric conditions.

### 4.4 Methodological and interpretative considerations

Several methodological and interpretative considerations should be acknowledged when evaluating the present synthesis.

First, sex distribution was not uniformly balanced across studies, and detailed sex-specific analyses were infrequently reported. Given accumulating evidence that gamma-band synchronization and excitation–inhibition balance may be modulated by biological sex and sex-steroid fluctuations (Anazawa et al., 2023, Jasinskyte et al., 2023, Hyer et al., 2018, Griskova-Bulanova et al., 2014, Melynyte et al., 2018), uneven sex ratios and the absence of control for hormonal status (e.g., menstrual cycle phase, contraceptive use, or menopausal status) may contribute to variability in reported ASSR–age relationships. Because such factors were rarely documented in the primary literature, their potential influence could not be systematically evaluated within the present review.

Second, although the synthesis focused primarily on age-related effects in healthy populations, a proportion of the included healthy samples were derived from control groups in clinical case–control studies. In such designs, matching procedures typically prioritize age and sex, whereas other demographic variables (e.g., educational level, socioeconomic status, or subclinical traits) are not consistently reported. As a result, the extent to which these factors may have influenced reported ASSR–age relationships remains unclear.

Third, variability existed across studies in participant state and task demands during ASSR recording. Recordings were conducted under passive listening (with or without distraction), active attention, or sleep conditions. Given that gamma-band synchronization is sensitive to arousal (Griskova et al., 2007, Wang et al., 2019) and attentional engagement (Matulyte et al., 2024), differences in recording context may have contributed to heterogeneous findings. However, systematic monitoring and reporting of vigilance state were infrequent, limiting cross-study comparability.

Fourth, methodological heterogeneity across stimulation parameters, recording configurations, and analytic approaches constrains direct comparison of findings. Variability in modulation frequency, carrier type, number of trials, sensor versus source analysis, and the selection of amplitude- versus phase-based metrics complicates efforts to delineate precise normative trajectories, and based on observed mixed results when different paradigms were used in the same study (Poulsen et al., 2007, Poulsen et al., 2009), could be a substantial source of variability. Although such diversity reflects the evolving nature of the field, it restricts the possibility of quantitative integration and standardization.

Fifth, most available evidence is cross-sectional. Inferred nonlinear lifespan patterns should therefore be interpreted cautiously, as cohort effects cannot be excluded. While cross-sectional comparisons provide important initial mapping, harmonized longitudinal paradigms spanning contiguous developmental and aging stages are needed to delineate trajectories of maturation, stabilization, and decline in gamma synchronization.

Finally, integration of ASSR measures with multimodal approaches, including structural imaging, magnetic resonance spectroscopy, and behavioral assessments, remains limited but may be helpful in clarifying the neurobiological mechanisms underlying lifespan changes in cortical synchronization.

Together, these considerations underscore the importance of improved methodological harmonization and comprehensive reporting. Such advances may support the development of standardized ASSR protocols suitable for both cross-sectional and longitudinal investigations, thereby strengthening the role of gamma-range ASSRs as a mechanistic biomarker across the human lifespan.

### 4.5 Generalization

Across the lifespan, the auditory gamma-range steady-state responses follow a non-linear developmental trajectory (Arutiunian et al., 2022, Cho et al., 2015, Edgar et al., 2016). When integrated with recent fetal evidence (Niepel et al., 2020), the available data now trace this trajectory from the late prenatal period through senescence, highlighting a lifespan continuum of oscillatory maturation and decline. During childhood and adolescence, 40-Hz ASSRs show progressive strengthening of amplitude and phase coherence, reflecting the maturation of auditory cortical circuits and refinement of excitation–inhibition balance (Candelaria-Cook et al., 2022, McKeon et al., 2023, Uhlhaas and Singer, 2010). This maturation appears to plateau by early adulthood, after which findings become more heterogeneous (Usui et al., 2023). In adulthood and aging, results diverge: some studies report attenuation of ASSR amplitude or phase consistency (Edgar et al., 2017; Griskova-Bulanova et al., 2013), others find enhanced responses (Dobri et al., 2023, Ross, 2018), and several observe no clear changes (Goossens et al., 2016, Tlumak et al., 2015). Such variability may stem from differences in individual variation in cortical plasticity, neurochemical balance, and compensatory recruitment (ElShafei et al., 2020, Murty et al., 2020) combined with methodological aspects.

Understanding lifespan changes in ASSRs has important translational implications. Establishing normative developmental and aging trajectories enables the differentiation of pathological alterations from age-appropriate variability, improving the interpretability of ASSR abnormalities in clinical contexts such as schizophrenia (Thuné et al., 2016, Zouaoui et al., 2023), autism spectrum disorder (Darrell et al., 2025, Seymour et al., 2020), and dementia (Mao et al., 2025, van Deursen et al., 2011). Moreover, by delineating when cortical synchronization is most plastic or vulnerable, lifespan data can inform the timing and personalization of interventions targeting gamma activity, whether through auditory or multi-modal stimulation (Chen et al., 2025, Olson, 2021), pharmacological modulation (Homma et al., 2025) or noninvasive brain stimulation (Mockevičius et al., 2025). Ultimately, integrating lifespan and clinical evidence will advance the use of ASSRs as a mechanistic biomarker for monitoring neural network integrity and treatment response across neurodevelopmental, psychiatric, and neurodegenerative conditions.

## CRediT authorship contribution statement

**Aurimas Mockevičius:** Writing – review & editing, Visualization, Validation, Methodology. **Danylo Machevskyi:** Writing – review & editing, Investigation. **Dariusz Majcherczyk:** Writing – review & editing, Investigation. **Inga Griškova-Bulanova:** Writing – review & editing, Writing – original draft, Visualization, Methodology, Conceptualization.

## Declaration of Generative AI and AI-assisted technologies in the writing process

During the preparation of this work, the authors used ChatGPT and Grammarly to improve language and readability. After using this tool/service, the authors reviewed and edited the content as needed and take full responsibility for the content of the publication.

## Declaration of Competing Interest

The authors declare that they have no known competing financial interests or personal relationships that could have appeared to influence the work reported in this paper.

## Data Availability

Data will be made available on request.
