# Supplementary material for: Developmental and aging trajectories of 40-Hz auditory steady-state responses: A systematic review across the human lifespan
Source: Dev Cogn Neurosci. 2026 Feb 5;79:101690. doi: 10.1016/j.dcn.2026.101690 (PMC12914391; doi:10.1016/j.dcn.2026.101690)
Supplement: Supplementary file 1 — Supplementary material [file mmc1.docx]

**Supplementary material.** Search keywords.

**Search in Pubmed**

Search 1

(("auditory steady-state response"[Title/Abstract] OR "auditory steady state response"[Title/Abstract] OR "steady-state auditory evoked potential"[Title/Abstract] OR "ASSR"[Title/Abstract] OR "SSAEP"[Title/Abstract])

AND

(child*[Title/Abstract] OR infant*[Title/Abstract] OR adolescent*[Title/Abstract] OR teen*[Title/Abstract] OR pediatric[Title/Abstract])

AND

(EEG[Title/Abstract] OR electroencephalography[Title/Abstract] OR MEG[Title/Abstract] OR magnetoencephalography[Title/Abstract]))

Search 2

(("auditory steady-state response"[Title/Abstract] OR "auditory steady state response"[Title/Abstract] OR "steady-state auditory evoked potential"[Title/Abstract] OR "ASSR"[Title/Abstract] OR "SSAEP"[Title/Abstract])

AND

(ageing[Title/Abstract] OR aging[Title/Abstract] OR "older adults"[Title/Abstract] OR elderly[Title/Abstract] OR aged[Title/Abstract] OR senescence[Title/Abstract])

AND

(EEG[Title/Abstract] OR electroencephalography[Title/Abstract] OR MEG[Title/Abstract] OR magnetoencephalography[Title/Abstract]))

Search 3

(("auditory steady-state response"[Title/Abstract] OR "auditory steady state response"[Title/Abstract] OR "steady-state auditory evoked potential"[Title/Abstract] OR "ASSR"[Title/Abstract] OR "SSAEP"[Title/Abstract])

AND

("age-related"[Title/Abstract] OR age[Title/Abstract] OR maturation[Title/Abstract] OR "lifespan"[Title/Abstract] OR "life span"[Title/Abstract] OR "development"[Title/Abstract] OR "age effect"[Title/Abstract] OR "age differences"[Title/Abstract])

AND

(EEG[Title/Abstract] OR electroencephalography[Title/Abstract] OR MEG[Title/Abstract] OR magnetoencephalography[Title/Abstract]))

**Search in Scopus**

Search 1

TITLE-ABS-KEY(("auditory steady-state response" OR "auditory steady state response" OR "steady-state auditory evoked potential" OR "ASSR" OR "SSAEP")

AND

(child* OR infant* OR adolescent* OR teen* OR pediatric)

AND

(EEG OR electroencephalography OR MEG OR magnetoencephalography))

Search 2

TITLE-ABS-KEY(("auditory steady-state response" OR "auditory steady state response" OR "steady-state auditory evoked potential" OR "ASSR" OR "SSAEP")

AND

(ageing OR aging OR "older adults" OR elderly OR aged OR senescence)

AND

(EEG OR electroencephalography OR MEG OR magnetoencephalography))

Search 3

TITLE-ABS-KEY(("auditory steady-state response" OR "auditory steady state response" OR "steady-state auditory evoked potential" OR "ASSR" OR "SSAEP")

AND

("age-related" OR age OR maturation OR "lifespan" OR "life span" OR development OR "age effect" OR "age differences")

AND

(EEG OR electroencephalography OR MEG OR magnetoencephalography))
